# Severe neurological impairment and immune function: altered neutrophils, monocytes, T lymphocytes, and inflammasome activation

**DOI:** 10.1038/s41390-024-03023-8

**Published:** 2024-01-17

**Authors:** John Allen, Johana Isaza-Correa, Lynne Kelly, Ashanty Melo, Aoife Mahony, Denise McDonald, Eleanor J. Molloy

**Affiliations:** 1 Discipline of Paediatrics, School of Medicine, Trinity College Dublin, the University of Dublin, Dublin, Ireland; 2 Trinity Research in Childhood Centre (TRiCC), Trinity College Dublin, the University of Dublin, Dublin, Ireland; 3 Trinity Translational Medicine Institute (TTMI) Trinity College Dublin, Dublin, Ireland; 4 Department of Neurodisability, Children’s Health Ireland (CHI) at Tallaght, Dublin, Ireland; 5 Department of Neonatology, CHI at Crumlin, Dublin, Ireland; 6 Coombe Hospital, Dublin, Ireland

## Abstract

**Background:**

Infections cause significant morbidity and mortality in children with Severe Neurological Impairment (SNI). Alterations in immune cell numbers and function in children with neurodisability have been reported. We aimed to characterise neutrophil, monocyte and lymphocyte proportions and activation, at baseline and in response to stimulation with lipopolysaccharide, in children with SNI compared to healthy controls.

**Methods:**

Whole blood samples of children with SNI and controls were incubated in the presence or absence of lipopolysaccharide (10 ng/ml). Monocyte and neutrophil function (Cluster of Differentiation (CD)11b, (TLR)-4 and CD66b expression) and lymphocytes were assessed by flow cytometry. Expression of genes involved in the inflammasome (NLR Family Pyrin Domain Containing(*NLRP)-3*, Apoptosis-Associated Speck-like protein *(ASC)* and Interleukin(*IL)1β*) were assessed by PCR.

**Results:**

Monocytes and CD8+ T cells were lower in children with SNI (*n* = 14). CD66b, was hyporesponsive and monocyte TLR4 was hyperresponsive to lipopolysaccharide in children with SNI compared to controls (*n* = 14). *NLRP3* expression was higher at baseline and *IL1β* expression was not upregulated in response to lipopolysaccharide in children with SNI in contrast to controls.

**Conclusion:**

We have found significant differences in immune regulation in children with SNI compared to controls which may provide a useful therapeutic target in the future.

**Impact:**

- Children with SNI have reduced monocyte and CD8+ T cells.
- Neutrophils and monocytes in children with SNI show altered markers of activation in response to lipopolysaccharide.
- Expression of *NLRP3* at the RNA level was higher at baseline in children with SNI.
- This study adds to the existing literature that children with neurological impairment have altered inflammatory and immune cell responses.
- This may provide a useful therapeutic target to reduce infection-related morbidity and mortality, and tertiary neurological injury in the future.

## Introduction

Children with Severe Neurological Impairment (SNI) have permanent disorders of the central nervous system which result in motor impairment, cognitive impairment, and medical complexity.^1^ In some cases, a child with SNI may not have a unifying diagnosis but all share the potential for dysfunction in almost every system as a comorbidity^2^ Some children with Cerebral Palsy (CP) would also fit the definition of SNI, but not all children with SNI have CP.

Infections, especially in the respiratory tract, are a major cause of morbidity and mortality in children with neurological impairment.^3^ This is most marked in those with the greatest motor impairment or the greatest total disability score,^3^ as would be seen in those with SNI. In a retrospective analysis of post-operative complications of appendectomy (*n* = 1250), Dhiman et al. have described significantly greater odds of sepsis/organ failure, operation-related infection, pneumonia, and urinary tract infection in children with CP compared to those without.^4^ The higher risk of infection in the population of children with neurodisability is likely multi-factorial including respiratory muscle weakness, poor clearance of secretions and aspiration contributing to respiratory tract infections.^5^ However, the immune dysfunction may have an important role in the development of significant infection-related morbidity and mortality.

Subclinical immunologic abnormalities have been found with γδT cell expansion in patients with neurological disorders, particularly CP^6^ and Taher et al have demonstrated alterations in T and B cell proportions, distributions, and functions in children with neonatal encephalopathy (NE) and in children with CP.^7^ Markers of granulocyte function, toll-like receptor (TLR)-4 are increased in preterm children with CP at school-age.^8^ Cytokine dysregulation is seen in childhood post NE and in children with CP.^9,10^

The inflammasome is a vital component of the innate immune response to infection and the nucleotide-binding and oligomerization domain (NOD)-like receptor protein 3 (NLRP3) is the most widely studied.^11^ The NLRP3 inflammasome has been implicated in numerous disease states,^12^ including neurological injury.^13^ Altered activation of the inflammasome has been shown to persist in childhood following NE.^12,14^ We aimed to characterise neutrophil, monocyte and lymphocyte proportions and activation, at baseline and in response to stimulation with lipopolysaccharide (LPS), in children with SNI compared to an age-matched control group of children.

## Methods

### Ethics and patient recruitment

Ethical approval was obtained from the Research Ethics Committee of Tallaght University Hospital, Dublin, Ireland (Ref: 2018/09 Chairman’s Action 7). Controls and children with SNI as previously defined^1^ were recruited from the outpatient department of a tertiary paediatric unit and written informed consent was obtained. Controls were healthy children, having phlebotomy performed for reasons unrelated to acute infection/inflammation or chronic illness. After informed consent was obtained, whole blood samples (3 mL) were collected in a sodium citrate anti-coagulated blood tubes. Samples where processed within 2 h of phlebotomy.

### Flow cytometry

100 µL aliquots of whole blood were incubated at 37 °C for 1 h, untreated (vehicle), or stimulated with LPS (E.coli 0111:B4: SIGMA Life Science, Wicklow, Ireland) 10 ng/mL. Fluorochrome -conjugated monoclonal antibodies (mAb) were used to stain the cells as follows: CD14-PerCP, CD15-PECy7, CD16- FITC, CD66b-Pacific Blue and TLR4-APC (BioLegend®, California; 2.5 µL/tube) and PE labelled CD11b (BD Biosciences, Oxford, UK; 10 μL per tube), γδ1-FITC, γδ2-PE, CD56-APC-Cy7, CD19-APC, CD4-PE-Cy7, CD8-PerCp. Red cells were lysed with 500 µL BD FACS™ 1X lysing solution (BD Biosciences, Oxford, UK). The prepared cells were acquired on a FACS CANTO II flow cytometer (BD Bioscience) and analysed using FlowJo Version 10 (Tree Star, Oregon) software. Neutrophils were delineated based on FSC-H and positive CD66b(+), monocytes on FSC-H and CD66b negativity (−) (Fig. S1). Relative expression of CD11b TLR4 and CD66b was expressed as mean channel fluorescence (MCF), which is the mean intensity of fluorescence emitted by all cells selected. Monocyte subsets were based on CD14 and CD16 expression: Classical (CD14+/CD16−), Intermediate (CD14+/CD16+), Non-classical (CD14dim/CD16+) (Fig. S1). Lymphocytes were selected based on SSC-A and FSC-A and subpopulations were defined as follows: B-cells (CD3-/CD19+), NK cells (CD3-/CD56+), T-cells (CD3+), CD4 (CD3+/CD4+), CD8 (CD3+/CD8+), γδ1 (CD3+/γδ1+), γδ2 (CD3+/γδ2+) (Fig. S2). Percentage change is used to express the relative change in markers of activation before and after LPS exposure as a proportion of the baseline value.

### Real time qPCR

Real time quantitative PCR was used to quantify the expression of three inflammasome-related genes i.e. NLRP3, ASC and IL1β. Whole blood was incubated at 37 °C for 1 h, untreated (vehicle), or stimulated with LPS (E.coli 0111:B4: SIGMA Life Science, Wicklow, Ireland) 10 ng/mL. RNA was extracted using the RiboPure™ blood kit (ThermoFisher Scientific, Waltham, MA) according to manufacturer instructions. The NanoDrop ND-100 Spectrophotometer was used to determine RNA purity and concentration using ND-1000 ver.3.1.2 software. TaqMan® primer probes were used to detect expression of the following inflammasome genes: NLRP3 (NM_001079821.2), ASC(NM_ 013258.4) and IL-1β(NM_000576.2). GAPDH (NM_002046.3) was used as an endogenous control for data normalisation. We employed the QuantStudio 5 real-time PCR system (ThermoFisher Scientific), and relative quantification values were calculated using the 2^−ΔΔCt^ method.^15^

## Results

### Participant characteristics

The 28 children recruited included 14 controls (7 male; mean age of 8.1 ± 4.5 years) and 14 children with SNI (7 male: a mean age of 11.4 ± 4.8 years). There were no statistically significant differences in age (*p* = 0.07) or sex (*p* > 0.99) between the two groups. In the children with SNI, nine had CP (four dyskinetic, three spastic, two mixed), two had Wolf-Hirschhorn syndrome, two had Rett syndrome and one had a Calcium/Calmodulin Dependent Serine Protein Kinase (CASK) mutation. In those with CP the aetiology was as follows: three had NE, two had infectious, two had prematurity, one had genetic, one had congenital brain malformation (Table S1).

### Effect of LPS endotoxin on neutrophils

**Fig. 1:**
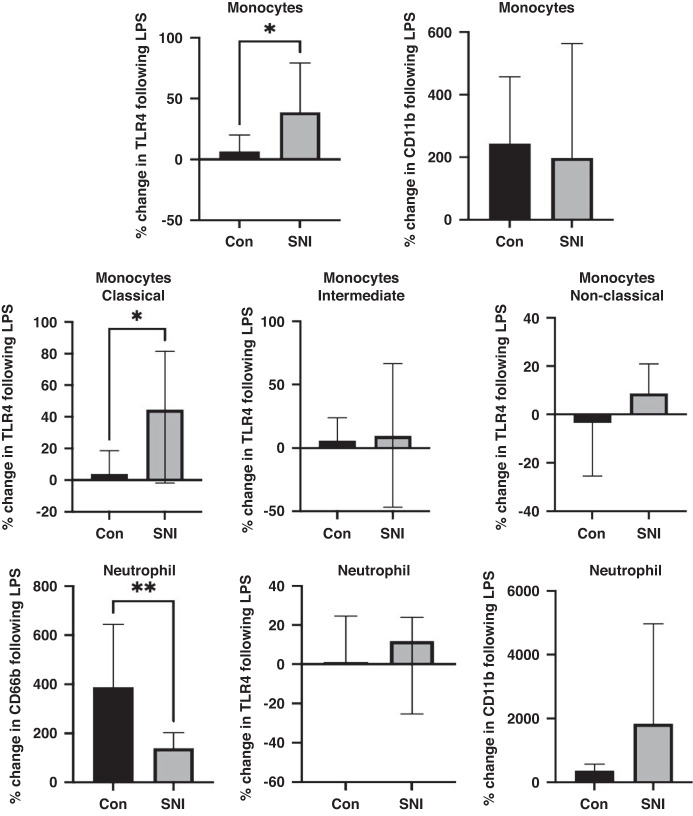
Percentage change in expression of markers of activation in monocytes, monocyte subsets and neutrophils following LPS. Whole blood samples were processed for flow cytometry and expression of CD66b, TLR4 and CD11b on neutrophils and monocytes was quantified at baseline and following LPS stimulation. Values are shown as percentage change in expression of CD66b, TLR4 and CD11b following incubation with LPS (10 ng/ml) for 1 h. Mann-Whitney test (median, 95%CI). Control (Con, *n* = 14); Severe Neurological Impairment (SNI, *n* = 14); *p ≤ 0.05; **p ≤ 0.01.

There was no difference in CD66b expression in either cohort at baseline (*p* = 0.33, Table S2, Fig. S3), implying similar numbers of neutrophils. Although both cohorts had significant increases in CD66b expression following LPS, children with SNI were relatively hyporesponsive, with a median percentage increase of 139% compared to 388% in the controls (*p* = 0.0017; Fig. 1).

Neutrophil TLR4 and CD11b expression were not significantly different at baseline or following LPS stimulation between the groups (Table S2, Fig. S3).

### Effect of LPS endotoxin on monocytes

Monocyte proportions at baseline were significantly lower in children with SNI when compared to controls (*p* = 0.0002, Table S2, Fig. S4) with no significant change in total monocyte proportions following LPS endotoxin stimulation in either group. However, there was a significant difference in the percentage change in monocyte TLR4 expression with LPS stimulation (*p* = 0.04) with the SNI cohort showing hyperresponsiveness (Fig. 1). At baseline and with LPS exposure, monocyte CD11b expression was no different between groups (Fig. 1, Table S2).

### Effect of LPS endotoxin on monocyte subsets

Monocytes were divided into their subsets based on positive CD14 and CD16, as previously outlined. Proportions of classical, intermediate or non-classical monocytes did not differ between the groups either before or after stimulation with LPS (Table S2, Fig. S5). However, in the control group, intermediate monocyte proportions reduced after LPS exposure (*p* = 0.04), unlike children with SNI (Fig. S5).

### Effect of LPS endotoxin on TLR4 and CD11b expression in monocyte subsets

Expression of TLR4 in classical monocytes did not differ at baseline or following LPS exposure between controls and children with SNI (Fig. 1). There was a relative hyperresponsiveness of TLR4 expression on classical monocytes following LPS exposure (*p* = 0.03; Fig. 1). There was no difference between groups in TLR4 expression before or after LPS exposure in intermediate or non-classical monocytes (Fig. 1). There was no difference between the groups in CD11b expression on any of the monocyte subsets either before or after LPS exposure (Fig. S5).

### Lymphocytes in children with SNI compared to controls

**Fig. 2:**
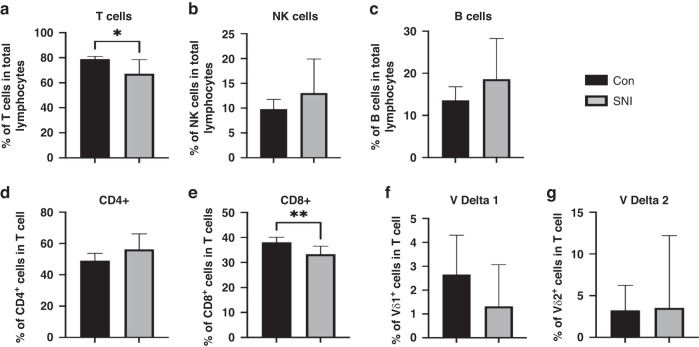
Proportion of T cells and CD8+ cells as a percentage of the total lymphocyte count are significantly lower in children with SNI compared to controls. T cells (**a**), NK cells (**b**), B cells (**c**), CD4+ (**d**), CD8+ (**e**), V delta 1 (**f**), V delta 2 (**g**). Samples were processed for flow cytometry. Values are expressed as percentages of total lymphocytes or T cells. Mann–Whitney test (median, 95% CI). Control (Con, *n* = 14); Severe Neurological Impairment (SNI, *n* = 14); NK natural killer cells, *p ≤ 0.05; **p ≤ 0.01.

T cells, as a proportion of total lymphocytes, and CD8+ T cells were significantly lower at baseline in children with SNI compared to controls (*p* = 0.02 & *p* = 0.003; Fig. 2 and Table S3). CD4+, γδ1+ and γδ2+ T cells, B cells and Natural Killer cells were not significantly different between the two groups (Fig. 2 and Table S3).

### Inflammasome

**Fig. 3:**
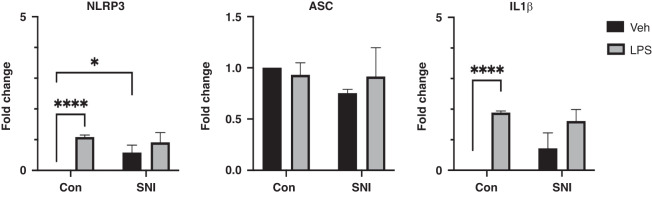
Expression of genes from the Nucleotide-binding and oligomerization domain (NOD)-like receptor protein 3 (NLRP-3) inflammasome at baseline and following LPS. Gene expression of *NLRP3*, Apoptosis-associated speck-like protein containing a CARD (*ASC*) and Interleukin 1β (*IL1β*), was quantified by real-time polymerase chain reaction (PCR) at baseline and following LPS. Values are expressed as fold changes in gene expression following incubation with LPS (10 ng/ml) for 1 h. Two way ANOVA (mean ± SD); Control (Con, *n* = 10); Severe Neurological Impairment (SNI, *n* = 5); Veh, Vehicle; *p ≤ 0.05; ****p ≤ 0.0001.

The NLRP3 inflammasome was evaluated in children with CP (*n* = 5) and compared with controls (*n* = 10). There was a significantly higher *NLRP3* gene expression at baseline in children with SNI compared to controls (*p* = 0.02; Fig. 3). On exposure to LPS, *NLRP3* expression was significantly upregulated in controls (*p* < 0.0001) but not in the SNI cohort (*p* = 0.50). Expression of *ASC* was not significantly different at baseline or following treatment with LPS between controls and children with SNI. *ASC* gene expression did not significantly increase following LPS exposure in either group. Expression of the *IL1β* gene was not significantly different at baseline between the cohorts but was significantly upregulated in response to LPS in controls (*p* < 0.0001) but not in children with SNI (*p* = 0.08; Fig. 3).

## Discussion

Expression of CD66b increased significantly in both children with SNI and controls following exposure to LPS. Children with SNI exhibited CD66b hyporesponsiveness compared to controls. CD66b is a glycosylated protein which is expressed by human neutrophils and can serve as a marker of activation in these cells.^16^ It plays a role in cell adhesion and migration in response to stimulation^17,18^ and significant upregulation of CD66b is seen in sepsis. Increased expression of CD66b is associated with neutrophil aggregation and cell adhesion.^17^ Cross-linking of the molecule leads to an oxidative burst and IL-8 release.^19^ Higher CD66b expression is thought to lead to more aggregate formation, more opportunity from cross-linking events and, therefore, increased adhesion of neutrophils.^17^ In adults, Kobold et al. found that reduced activation of both monocytes and neutrophils at diagnosis of sepsis correlated with higher mortality.^20^ Weinshenk et al. demonstrated that preterm infants with culture-proven or suspected sepsis had increased CD66b expression on leukocytes which did not correlate with clinical outcome.^21^ CD66b hyporesponsiveness may translate as increased morbidity and mortality related to infection.

Monocytes were reduced in children with SNI compared to controls. Monocytes are agranulocytes which function as part of the innate immune system through phagocytosis, antigen presentation and cytokine production. It has been proposed that classical cells, mobilised in response to inflammation, differentiate to non-classical monocytes, with intermediate monocytes acting as a transitional phase.^22^ Other authors advocate that intermediate monocytes represent a distinct subset, with their own discrete function.^23^ However monocytes are essential in early response to tissue injury and in bridging the gap between the innate and adaptive immune systems.^23,24^ In adults with sepsis, Chung et al. reported that lower monocyte counts were associated with higher mortality, rate of bacteraemia, mechanical ventilation, vasopressor use and renal replacement therapy.^25^

In this study, we have shown that the monocytes of children with SNI had relative hyperresponsiveness of TLR4 expression when exposed to LPS in comparison to controls. This relative hyperresponsiveness was seen in the classical, but not in the intermediate or non-classical monocyte subsets. Classical monocytes are considered strongly pro-inflammatory and exhibit superior phagocytosis.^23^ This may indicate a pro-inflammatory state in children with SNI, as has been previously been suggested by the work of Zareen et al. in children with NE and CP^9,10^ where altered cytokine responses were noted following exposure to LPS.

A significant reduction in total T cells was observed in children with SNI in comparison to controls. T cells play a crucial role in host defence against viruses and intracellular bacteria as well as regulating the inflammatory response. Therefore, any alteration in T lymphocyte proportions or function may contribute to inflammatory dysregulation or a reduced capacity to respond to infection. A reduction in CD8+ cytotoxic T cells in children with SNI was also seen. These T cells play an important role in host defence against intracellular pathogens, such as viruses and intracellular bacteria, by recognising peptides bound to Major Histocompatibility Complex Class 1 found on all nucleated cells. Future work will include looking in more detail at elements of CD8 T cell function, such as cytotoxicity and regulatory profile.

In this study, the expression of *NLRP3* and *IL1β* increased significantly in response to LPS stimulation in controls but not in children with SNI. However, baseline *NLRP3* expression was higher in children with SNI compared to controls. The results presented here represent *NLRP3* gene component expression and may not necessarily correspond to activation of the complex at protein level. Future work will seek to further clarify whether alterations at the RNA level translate into changes downstream, e.g., by analysing caspase-1 activation and IL1β secretion. However, our results may represent background low-grade inflammation and trained immunity, in which prior exposure to LPS causes the cells of the innate immune system to become less responsive to further challenges. This may be advantageous in preventing conditions with an exaggerated inflammatory response, such as the cytokine storm which may be seen in sepsis and is associated with increased mortality.^26^ However, there may be a reduction in the innate inflammatory response to infection indicating a relative immunosuppression.^12^ The NLRP3 inflammasome is an essential component of the innate immune system. It is expressed in neutrophils, monocytes, and lymphocytes.^27^ The NLRP3 inflammasome consists of a sensor (NLRP3), an adaptor (Apoptosis-associated speck-like protein containing a CARD; ASC) and an effector (Caspase-1), the activation of which leads to secretion of the pro-inflammatory cytokines, IL1β and IL18.^11^ NLRP3 inflammasome assembly also causes cleavage of gasdermin D (GSDMD), triggering pyroptosis (a highly inflammatory form of programmed cell death).^11^ GSDMD exhibits bactericidal activity, contributing to the innate immune response to infection.^11^ The NLRP3 inflammasome has been implicated in numerous disease states including inflammatory bowel disease,^28^ neurodegenerative disorders^29^ and sepsis.^30^ It has generated considerable interest as a potential therapeutic target for many disorders in which it has been proposed to play a role.^31^ In neonates, dysregulation of NLRP3 has been demonstrated in NE and this dysregulation persists in childhood.^12^ Abnormalities in innate immune signalling have been linked with many neurodevelopmental disorders, including autism and schizophrenia.^32^

There is a significant paucity of research in the area of immune and inflammatory dysregulation in children with neurodisability. A limitation of this study is the relatively small sample size. However, this study raises immune dysregulation as a potential contributory factor to the increased infection-related morbidity and mortality in SNI. A potential mechanism for these increased risks is endotoxin tolerance, which describes a refractory state in which an initial exposure to endotoxin renders the innate immune system temporarily unable to mount a subsequent response.^33^ Endotoxin tolerance does not just occur following infective processes but has been shown to occur following tissue damage from other sources such as myocardial infarction,^34^ in a process known as “heterotolerance”. Endotoxin tolerance is associated with increased risk of secondary infections.

Alterations in immune function have the potential to, at least partially, explain the increased burden of infection-related morbidity and mortality in this population. In addition, the cells of the immune system are central to initiating, maintaining and abolishing the inflammatory response. In health, pro- and anti-inflammatory influences are finely balanced and dysfunction can lead to abnormal response to infectious stimuli,^35^ multi-organ dysfunction,^36^ auto-immunity, and tertiary neurological damage.^37^ We have described altered proportions of a number of lymphocyte and granulocyte sub-populations in children with SNI. We have also illustrated alterations in the response of neutrophils and monocytes when exposed to the endotoxin LPS.

Children with CP and those who have had neonatal encephalopathy (NE) are known to have persistent inflammation into school-age.^9,10^ It is speculated that this persistent inflammation may lead to tertiary brain damage, in particular, if exposed to a second or subsequent “hit”.^37^ Lymphocytes are likely to play a key role in this inflammatory dysregulation. There may be some parallels with the phenomenon in older individuals termed “inflammaging” in which chronic overstimulation of the innate immune system leads to an ongoing, low-grade inflammation. This has been associated with significant increases in morbidity and mortality.^38^

Our findings suggest that there may be an element of immune dysregulation in children with SNI compared to controls and they add to the small amount of existing literature which have pointed to the same conclusion. However, further in-depth analysis into inflammasome activation at the protein level, cell phenotyping, distribution and function are required, as well as future prospective longitudinal studies with larger patient numbers which correlate these differences with health-related outcomes. It may then be possible to suggest specific immunomodulatory therapies which may be useful in improving health-related outcomes in children with SNI.

### Supplementary information

Supplementary information

Table S2

## Data Availability

The datasets generated during and/or analysed during the current study are available from the corresponding author upon reasonable request.
