# Supplementary material for: Severe neurological impairment and immune function: altered neutrophils, monocytes, T lymphocytes, and inflammasome activation
Source: Pediatr Res. 2024 Jan 17;95(6):1611–6. doi: 10.1038/s41390-024-03023-8 (PMC11126379; doi:10.1038/s41390-024-03023-8)
Supplement: Supplementary file 1 — Supplementary information [file 41390_2024_3023_MOESM1_ESM.pdf]

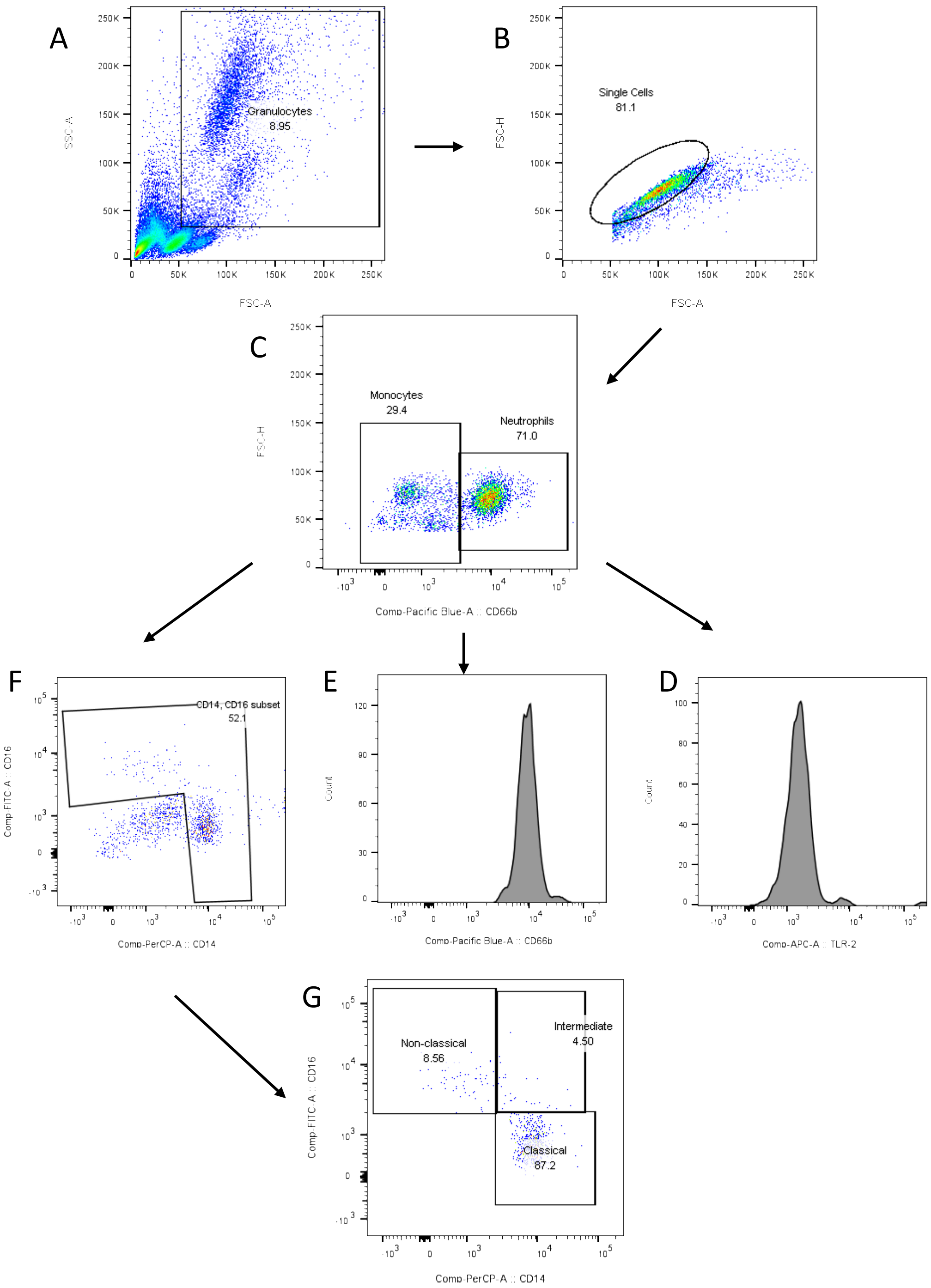

Figure S1. Gating strategy for Granulocytes. A) Flow cytometry dot plot showing forward scatter (FSC-A) and side scatter (SSC-A) and gated granulocytes. B) Doublets excluded by gating on single cells in FSC-H and FSC-A flow cytometry dot plot. C) Flow cytometry dot plot showing gated neutrophils and monocytes. D) Mean fluorescence intensity (MFI) of Toll-Like Receptor 4 (TLR-4) positive neutrophils. E) MFI of Cluster of Differentiation (CD)-66b positive neutrophils. F) Flow cytometry dot plot showing gated CD14 and CD16 positive subsets of monocytes. G) Flow cytometry dot plot showing gated classical, intermediate and non-classical monocytes.

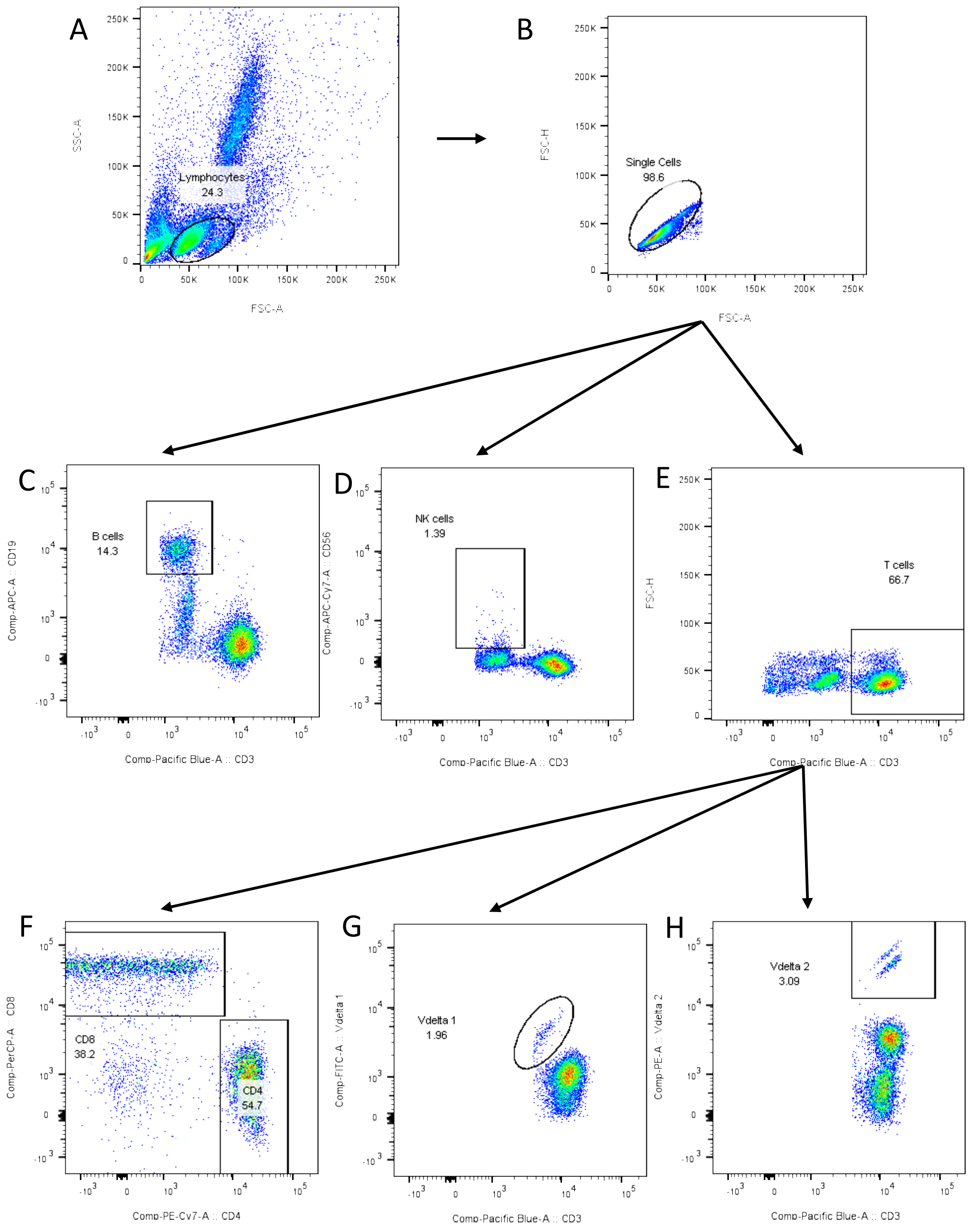

Figure S2. Gating strategy for lymphocytes. A) Flow cytometry dot plot showing forward scatter (FSC-A) and side scatter (SSC-A) and gated lymphocytes. B) Doublets excluded by gating on single cells in FSC-H and FSC-A flow cytometry dot plot. C) Flow cytometry dot plot showing gated B cells. D) Flow cytometry dot plot showing gated Natural Killer (NK) cells. E) Flow cytometry dot plot showing gated T cells. F) Flow cytometry dot plot showing gated Cluster of Differentiation (CD)-4 positive T cells. G) Flow cytometry dot plot showing gated V-delta-1 T cells. H) Flow cytometry dot plot showing gated V-delta-2 T cells.

Table S1. Clinical characteristics of children in the SNI group. SNI, Severe Neurological Impairment; CP, Cerebral Palsy; CASK, Calcium/Calmodulin Dependent Serine Protein Kinase; GMFCS, Gross Motor Function Classification System; VNS, Vagal Nerve Stimulator; RTI, Respiratory Tract Infection; PEG, Percutaneous Endoscopic Gastrostomy; PEJ, Percutaneous Endoscopic Jejunostomy.

**Variable**	**n**
**Diagnosis**	
CP	9
-Dyskinetic	*4*
-Spastic	*3*
-Mixed	*2*
Wolf Hirschhorn Syndrome	2
Rett syndrome	2
CASK mutation	1
**Aetiology of CP**	
Neonatal Encephalopathy	3
Congenital brain malformation	2
Infection	2
Prematurity	2
Genetic	1
**GMFCS (%)**	
I-III	0
IV-V	14
**Intellectual disability**	14
**Epilepsy**	11
Requiring 0-2 anti-epileptic medications	*5*
Requiring >2 anti-epileptic medications/VNS	*6*
**Visual Impairment**	8
**Hearing Impairment**	3
**Recurrent RTI requiring prophylaxis**	4
**Respiratory supportive technology**	5
**Feeding route**	
Oral	5
PEG	8
PEJ	1
**Number of regular medications;** *mean (SD)*	6.64 (3.57)

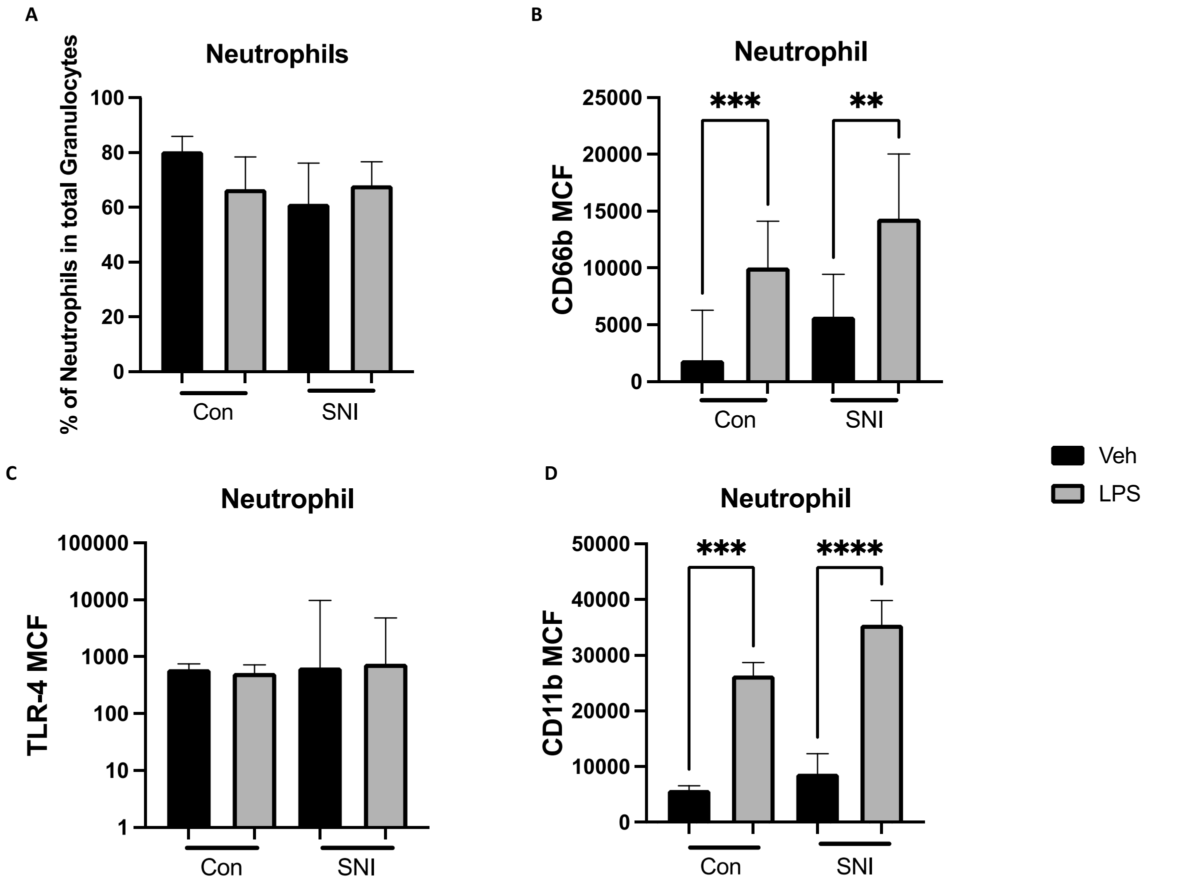

Figure S3. Neutrophil proportions and markers of activation at baseline and following LPS

Whole blood samples were processed for flow cytometry and expression of CD66b, TLR4 and CD11b on neutrophils was quantified following lipopolysaccharide (LPS). Neutrophil proportions are expressed as a percentage of total granulocyte numbers; values for CD66b, TLR4 and CD11b are expressed as Mean Channel Fluorescence (MCF). Kruskal Wallis test (median, 95%CI); Control (Con, n=14); Severe Neurological Impairment (SNI, n=14); Vehicle (Veh); **p£0.01; ***p£0.001; ****p£0.0001; (A) Proportion of neutrophils in total granulocytes; (B) Neutrophil CD66b expression; (C) Neutrophil TLR4 expression; (D) Neutrophil CD11b expression

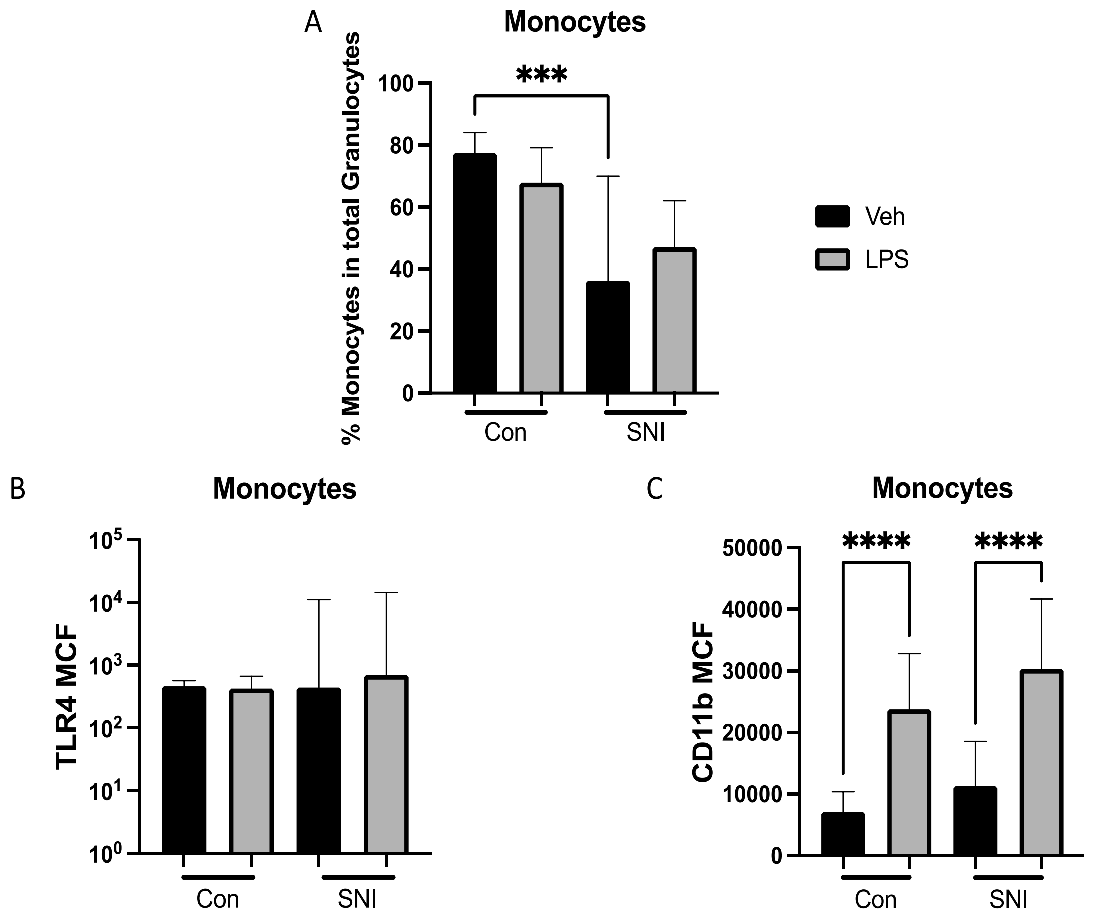

Figure S4. Monocyte proportions and markers of activation at baseline and following LPS

Whole blood samples were processed for flow cytometry and expression of TLR4 and CD11b on monocytes was quantified. Proportion of monocytes are expressed as a percentage (%) of total granulocytes. Values for TLR4 and CD11b are expressed as Mean Channel Fluorescence (MCF). Kruskal-Wallis test (median, 95%CI). Control (Con, n=14); Severe Neurological Impairment (SNI, n=14); Vehicle (Veh); **p£0.01; ***p£0.001; ****p£0.0001; (A) Proportion of monocytes in total granulocytes; (B) Monocyte TLR4 expression; (C) Monocyte CD11b expression.

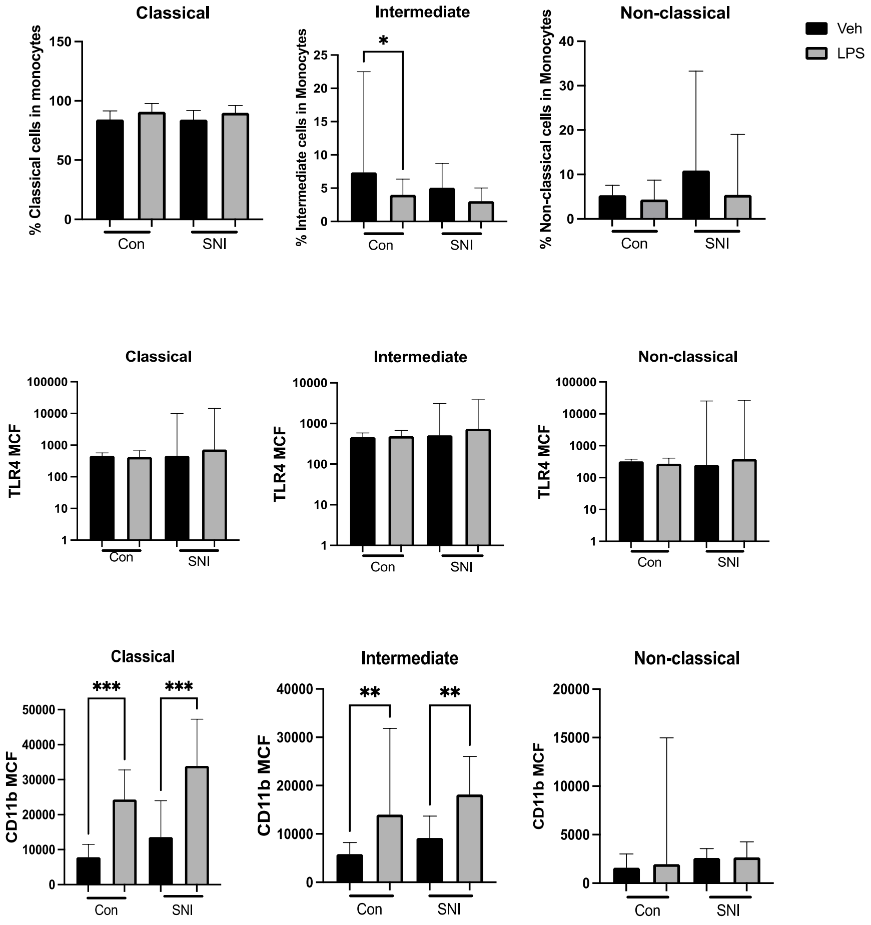

Figure S5. Monocyte subsets as proportions of total monocytes, and their expression of TLR4 and CD11b before and after LPS

Whole blood samples were processed for flow cytometry. Proportion of monocyte subsets are expressed as a percentage (%) of total monocytes and their expression of TLR4 and CD11b are expressed as Mean Channel Fluorescence (MCF). Kruskal-Wallis test (median, 95%CI). Control (Con, n=14); Severe Neurological Impairment (SNI, n=14); *p£0.05.

Table S3. T cell subset proportions as a percentage of total T cells. Whole blood samples were collected from controls (n=14) and children with Severe Neurological Impairment (SNI; n=14). Samples were processed for flow cytometry CI, Confidence Interval. **p£0.01

	**Control**		**SNI**		
**T cell subset**	Median	95% CI	Median	95% CI	p value
**CD4+**	49.05	45.70-53.60	56.20	48.30-66.10	0.05
**CD8+**	38.00	35.50-40.00	33.30	26.30-36.50	**0.0031****
**gd1+**	2.65	1.48-4.30	1.32	0.94-3.07	0.08
**gd2+**	3.25	1.45-6.24	3.55	1.32-12.20	0.70
